# Evaluation of Ride Comfort in a Railway Passenger Car Depending on a Change of Suspension Parameters

**DOI:** 10.3390/s21238138

**Published:** 2021-12-06

**Authors:** Ján Dižo, Miroslav Blatnický, Juraj Gerlici, Bohuš Leitner, Rafał Melnik, Stanislav Semenov, Evgeny Mikhailov, Mariusz Kostrzewski

**Affiliations:** 1 Department of Transport and Handling Machines, Faculty of Mechanical Engineering, University of Žilina, Univerzitná 8215/1, 010 26 Žilina, Slovakia; miroslav.blatnicky@fstroj.uniza.sk (M.B.); juraj.gerlici@fstroj.uniza.sk (J.G.); 2 Department of Fire Engineering, Faculty of Security Engineering, University of Žilina, St. 1. Mája, 010 26 Žilina, Slovakia; bohus.leitner@fbi.uniza.sk; 3 Faculty of Computer Science and Food Science, Lomza State University of Applied Sciences, Akademicka 1, 18-400 Łomża, Poland; rmelnik@pwsip.edu.pl; 4 Department of Logistics and Traffic Safety, Educational and Scientific Institute of Transport and Building, Volodymyr Dahl East Ukrainian National University, Central Avenue 59A/403, 93400 Severodonetsk, Ukraine; semenov@snu.edu.ua (S.S.); mihajlov@snu.edu.ua (E.M.); 5 Division of Construction Fundamentals of Transport Equipment, Faculty of Transport, Warsaw University of Technology, St. Koszykowa 75, 00-662 Warsaw, Poland; mariusz.kostrzewski@pw.edu.pl

**Keywords:** ride comfort, acceleration signals, rail vehicle, multibody simulation

## Abstract

Ride comfort for passengers remains a pressing topic. The level of comfort in a vehicle can influences passengers’ preferences for a particular means of transport. The article aims to evaluate the influence of changes in suspension parameters on the ride comfort for passengers. The theoretical background includes a description of the applied method for a creating the virtual model of an investigated vehicle as well as the method of evaluating the ride comfort. The ride comfort of the vehicle is assessed based on the standard method, which involves calculating the mean comfort method, i.e., ride comfort index *N_MV_* in chosen points on a body floor. The *N_MV_* ride comfort index (Mean Comfort Standard Method) requires the input of acceleration signals in three directions. The rest of the article offers the results of simulation computations. The stiffness–damping parameters of the primary and secondary suspension systems were changed at three levels and the vehicle was run on the real track section. The ride index *N_MV_* was calculated for all three modifications of the suspension system in the chosen fifteen points of the body floor. It was found that lower values in the stiffness of the secondary suspension system lead to lower levels of ride comfort in the investigated railway passenger car; however, lower values in the stiffness–damping parameters of the primary suspension system did not decrease the levels of ride comfort as significantly.

## 1. Introduction

A vehicle is a complex mechanical system with specific properties. In term of mechanics, it is a multibody system, the behavior of which can be analyzed based on valid criteria regarding the track and the surrounding environment [1,2,3,4,5].

The process of designing a vehicle has to meet specific requirements and operational conditions. These requirements mainly include running safety and ride comfort for passengers and they are described, verified and tested according to standards [6,7,8,9].

The main purpose of this work is to analyze the properties of a railway passenger car by means of simulation computations performed in a commercial simulation software. For these purposes, models of a railway passenger car and a track are created based on the available data. The research examines how changes in the railway passenger car parameters and operational conditions influence the ride comfort for passengers. The evaluation of ride comfort for passengers requires knowledge of the acceleration of given locations of a railway passenger car body [10,11,12,13,14]. The vehicle model is created in the Simpack software package (Dassault Systèmes, Vélizy-Villacoublay, France). The software library contains a measuring element, an accelerometer, which serves the purpose of measuring acceleration. After performing simulation computations, the acceleration signal is processed in the software PostProcessor. As a result, ride comfort indices are quantified.

The current state of simulation computations allows precise estimations and predictions of the dynamic properties of a vehicle in real operational conditions [15,16,17,18,19,20,21]. It is important to set up a virtual model with characteristics representing real states as accurately as possible. In the simulation software, the multibody system of the railway passenger car consists of rigid bodies interconnected by spring-damping elements [22,23]. The analyzed model of a vehicle includes the set of acceleration sensors, i.e., accelerometers, which measure the acceleration of the vehicle body in all three directions [24]. These acceleration signals are essential when calculating the ride comfort indices, based on which the ride comfort for passengers is assessed [25,26,27].

## 2. Motivation and the Research Goal

The authors of the presented research have for a long time devoted themselves to investigating the running properties and behaviors of wheeled means of transport. Their research is focused mainly on ride safety, passenger ride comfort and ways to improve them [28,29,30]. For these purposes, they have created many simulation models, performed a large number of computer simulations and undertaken investigations by means of test stands [31,32]. Their research deals mainly with rail vehicles [28,33,34,35] and road vehicles [36,37,38,39,40]. During these activities, various phenomena have been observed. Among them, it has been observed that it is possible to signify decreased running safety or running comfort by investigating changes in the parameters of vehicles, such as: mass and inertia parameters, parameters of suspension systems, the design of contact elements (the wheel/rail contact), the influence of input parameters, including the excitation of a vehicle’s mechanical system, stochastic phenomena during the running of a vehicle on a track and other effects [29,30,41].

This work presents and concludes selected findings about the influence of changes in the stiffness–damping parameters of a suspension system of a railway passenger car on passenger ride comfort. The method for evaluating ride comfort is based on the EN 12299:2009 standard [42].

## 3. Ride Comfort for Passengers

The analysis of ride comfort for passengers plays an essential role in the process of assessing vehicles. Ride comfort is a complex term, which includes a set of factors specifying thermal comfort, acoustic comfort [43,44], air quality and, in particular, the vibration of passengers [45,46,47]. Sensations related to ride comfort also depend on factors which characterize the environment in which a passenger is placed. Among other factors, the position of a passenger, the operational conditions and the time of exposure are all included. Despite the fact that the ride comfort level is closely related to these described factors, a quantitative evaluation of ride comfort is relatively difficult to achieve because of the subjective nature of the perception of various effects. The influence of various factors which relate to ride comfort is investigated by means of approximate methods describing their effects on a human body. Ride comfort for passengers is assessed by means of indices of ride comfort, which depend on one or more parameters characterizing the environment in which a passenger is located [41,47,48,49].

The above factors all have an effect on ride comfort, but by far the most important are vibrations and oscillations acting on the human body. These can influence in various ways and even worsen the organic functions of a person, can increase exhaustion and can even lead to health damage. When it comes to evaluating vehicle vibrations, total vibrations are important, i.e., vibrations which are transmitted from a seat or a floor to the passenger’s entire body [48,49,50].

The quantification of ride comfort for passengers is evaluated either by a direct method, i.e., by means of evaluating the comfort of a real vehicle, or by an indirect method, i.e., by means of evaluating specific measured data. The indirect method is based on the knowledge of acceleration signals relating to specific locations on a vehicle. Acceleration signals are filtered and weighted by functions which take into account the sensitivity of the human body in response to vibrations in various directions [48,51,52,53].

**Figure 1:**
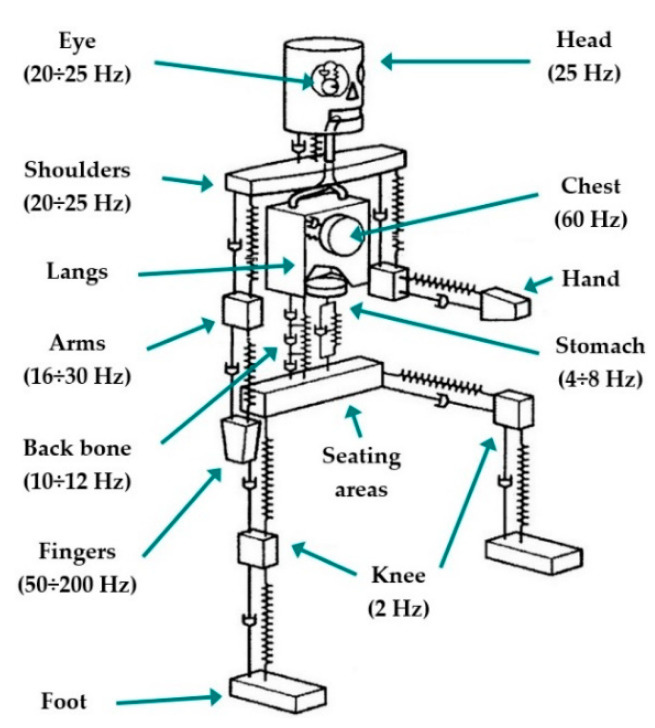
A dynamic model of a human body [48,49].

When vibrations act on a human body, forced oscillations of spcific parts of the body or the entire body are excited. In particular, this depends on both how the vibrations are transmitted to the human body and on the physical properties of the oscillations (i.e., intensity and frequency). When the excitation frequency is close to the eigenfrequencies of important human organs, severe consequences can occur. In the case of vertical oscillations, the eigenfrequencies of a human body range from 4 to 6 Hz. In this frequency range, resonance oscillations of the upper part of a body as well as the vertebrae and stomach are reached. In a horizontal direction, the resonance oscillation occurs in the range of 1 to 3 Hz. Generally, the human body can accept higher levels of horizontal oscillation than vertical oscillation [48,49]. A dynamic model of a human body is shown in Figure 1.

Signals of accelerations acting to a human body are the main parameters, based on which the ride comfort for passengers regarding vibrations is determined and evaluated.

### 3.1. Evaluation of Ride Comfort for Passengers

The sensations which a passenger experiences during the running of a vehicle due to vibrations are measured and assessed by means of the EN 12299 standard [42] and are recognized as the following [42,54,55,56,57]:

- Average comfort: average lasting feeling evaluated by means of the indices *N_MV_* (Mean Comfort Standard Method), *N_VA_* and *N_VD_* (Mean Comfort Complete Method);
- Continuous comfort: sensations evaluated during a certain time period by means of the indices *C_Cx_*, *C_Cy_* and *C_Cz_* (Continuous Comfort);
- Comfort during rail vehicle running in a curve: discomfort experienced during entering a curve or experienced during running in opposite curves by means of the index *P_CT_* (Comfort on Curve Transitions);
- Comfort during discrete events: discomfort experienced based on transient vibrations in a straight track and in curves for the index *P_DE_* (Comfort on Discrete Events).

This research is focused on the investigation and evaluation of average ride comfort for passengers measured on a rail vehicle floor by means of the index *N_MV_*. Details of the application of this index for evaluating the ride comfort for passengers are listed in Table 1.

Signals of measured accelerations in individual directions *x*, *y* and *z* (i.e., accelerations *a_x_*, *a_y_* and *a_z_*) are entered into the ride comfort calculation.

A local coordinate system is placed on a vehicle floor and it is defined by:

- An origin: in the middle of bogies pivots, the *x* axis for a longitudinal direction, the *y* axis for a lateral direction and the *z* axis for a vertical direction;
- Angle *φ*: the definition of rotary motion around the x axis.

#### Calculation of the Index N_MV_

The index *N_MV_*, calculated by a standard method, quantifies the ride comfort level on a rail vehicle floor for a sitting passenger. It is necessary to know the values of acceleration in the longitudinal (*x* axis), lateral (*y* axis) and vertical (*z* axis) directions. Values of acceleration are subsequently weighted by the weight functions *W_b_* and *W_d_* a frequency range of 0.4 to 100 Hz [42,47,55].

**Figure 2:**
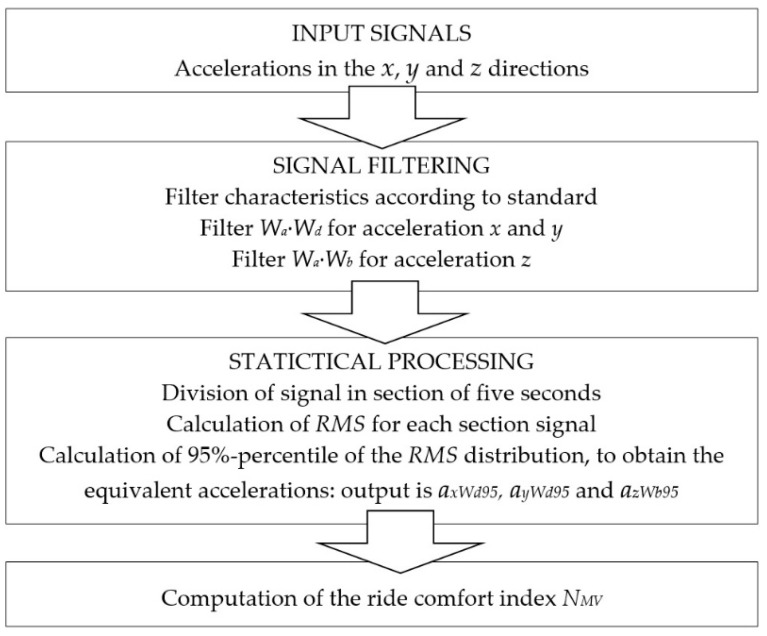
The calculation process for the ride comfort index *N_MV_*.

The mean square value of these accelerations are calculated as follows [42,58,59]:

(1) $$a_{j\eta }^{Wi}\left(t\right)=\sqrt{\frac{1}{T}\cdot \int_{t-T}^{T}{\left[{\ddot{x}}_{W_{i}}^{*}\left(\tau \right)\right]}^{2}\cdot d\tau },\;j=x,y,z,$$

where *T* = 5 s and *t* is a multiple of 5 s. Modified values of accelerations are statistically evaluated in the corresponding directions and a summation function is determined. Finally, 95%-percentiles of the distribution function in 5 s intervals are estimated from histograms. The calculation process of the ride comfort index *N_MV_* is depicted in Figure 2.

The resulting values of the ride comfort index *N_MV_* is given by the formulation [42]:

(2) $$N_{MV}=6\cdot \sqrt{{\left(a_{xP95}^{W_{d}}\right)}^{2}+{\left(a_{yP95}^{W_{d}}\right)}^{2}+{\left(a_{zP95}^{W_{b}}\right)}^{2}}.$$

A specific calculated value of the ride comfort index *N_MV_* is compared with the scale introduced in Table 2 for the final determination of the ride comfort in the given operational conditions.

### 3.2. The Method of Evaluation of Output Signals from Simulation Computations

The results of dynamic analyses of the investigated railway passenger car with the defined parameters and under the defined operational conditions are described below.

**Figure 3:**
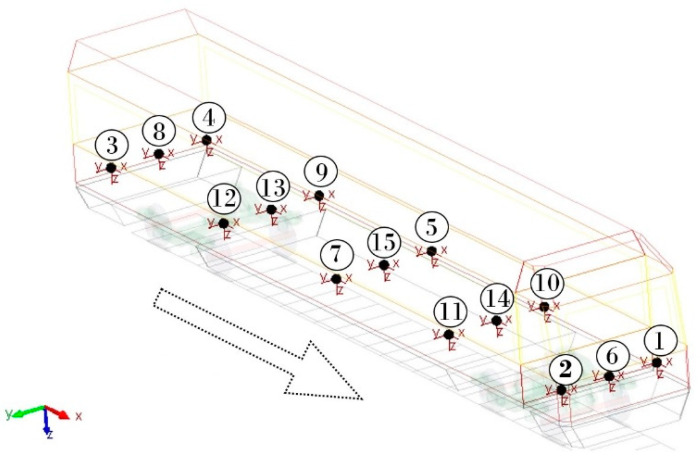
Locations of points for evaluation of ride comfort for passengers on the body floor.

As the main criterion, the level of ride comfort for passengers of a railway passenger car is evaluated based on the values of the ride comfort indices. Acceleration signals in three directions, *x*, *y* and *z*, are the main inputs for their calculation. Fifteen locations are chosen on the car body floor from which acceleration signals are measured. These points are shown in Figure 3.

**Figure 4:**
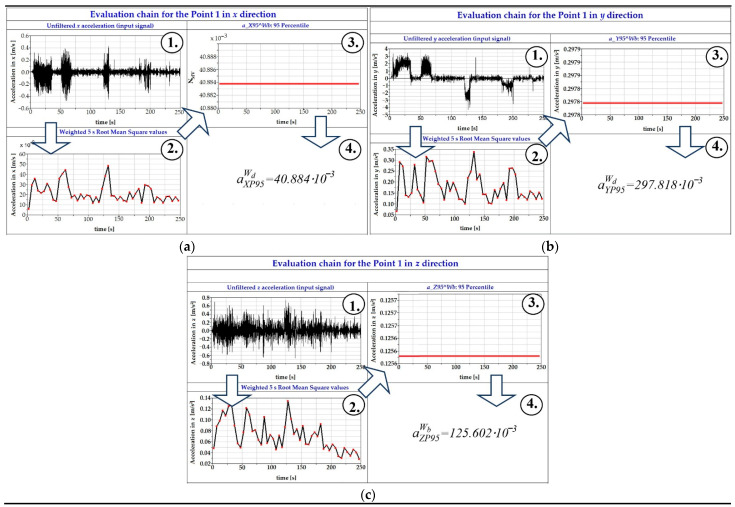
The procedure of processing acceleration signals in the Simpack software postprocessor: (**a**) for the *x* direction; (**b**) for the *y* direction; (**c**) for the *z* direction.

Acceleration signals have to be processed according to the valid standard [42,58]. Details are described in the following section. The software used includes a database with filters, which are applied to the obtained acceleration signals. The procedure of signal processing consists of several steps, which are shown in Figure 4.

Figure 4 has three individual parts for every direction of acceleration, i.e., the *x* direction, the *y* direction and the *z* direction.

## 4. Analysis of Ride Comfort of a Railway Passenger Car by Means of the Standard Method

**Figure 5:**
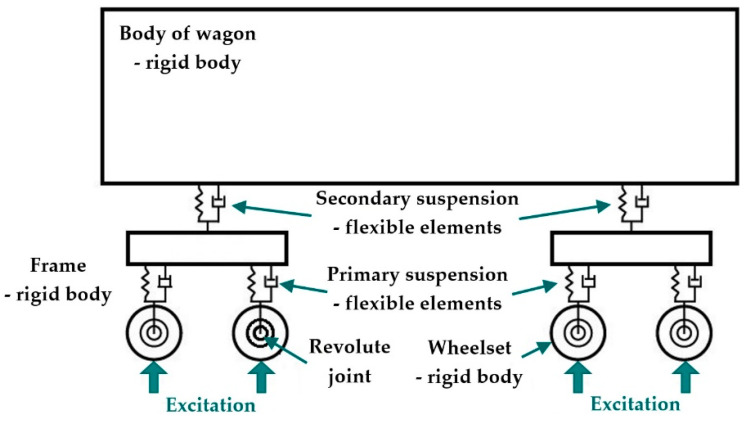
A scheme of the investigated railway passenger car.

When one uses commercial computational software for creating a virtual model of a vehicle, a mathematical model of a multibody system is generated automatically by the software. The number of equations depends on the complexity of the multibody system. The analyzed railway passenger car consists of rigid bodies and each of them has six degrees of freedom in the space. Couples between bodies restrict some of degrees of freedom. A scheme of the railway passenger car is shown in Figure 5.

The considered computational model of the railway passenger car includes mechanical as well as kinematic couples. Its dynamics are described by means of differential-algebraic equations [60,61,62], which are generally formed as follows:

(3) $$\left[\begin{array}{cc} M & D^{T}\\ D & 0 \end{array}\right]\cdot \left[\begin{array}{c} q\\ \Lambda \end{array}\right]=\left[\begin{array}{c} F\\ \gamma \end{array}\right]$$

where ***M*** is the mass matrix, ***D*** is the Jacobian matrix, ***q*** is the vector of generalized coordinates, ***Λ*** is the vector of Lagrange multipliers, ***F*** is the load vectors (including kinematic excitation, external forces, etc.), and $\gamma =D\cdot \ddot{q}$.

The dynamic analysis of a vehicle is required to define initial conditions, e.g., a position vector and/or a velocity vector. Based on this information, Equation (1) is set up and solved for the desired accelerations, which are subsequently integrated in time together with velocities.

### 4.1. A Description of a Multibody Wagon Model

A multibody model of the referenced rail vehicle was chosen for performing simulation computations. It is a two bogies four-axle railway passenger car. Wheelsets are guided in a bogie by means of two swinging arms and they are suspended by coil springs and hydraulic dampers on a frame. Yaw dampers eliminate the rotary motions of a bogie against the body of the wagon. A secondary suspension between bogie frames and the wagon body consists of coil springs and hydraulic dampers. Additional dampers are mounted in a lateral direction in order to reduce tilting of the body [63].

The wagon model was set up in the Simpack multibody software package. This software is a commercial multibody simulation software used for simulating non-linear motion of large and complex multibody systems.

**Figure 6:**
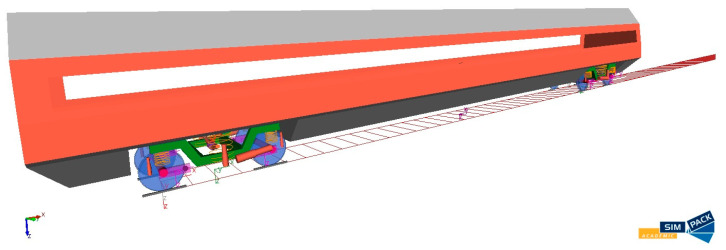
An illustration of the multibody wagon model created in the Simpack package.

In terms of the computational model, the created wagon model is composed of rigid bodies which are connected by massless spring-damper elements. An illustration of the multibody wagon model is shown in Figure 6.

The assembly of the wagon in the Simpack package includes three subsystems (in Simpack called substructures):

- A front bogie;
- A rear bogie;
- A wagon body.

**Figure 7:**
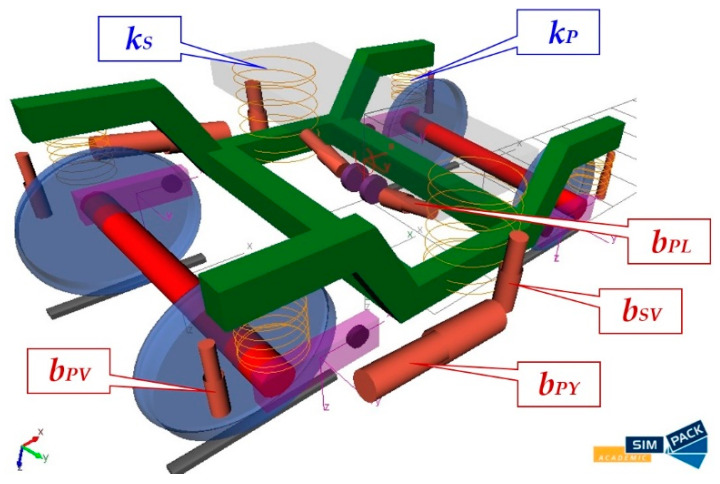
An illustration of the created multibody model of the bogie with designated individual suspensions components.

The bogie of the wagon with marked individual components of the primary and secondary suspension system is depicted in Figure 7.

The designation of springs and dampers is as follows:

- *k_P_*—stiffness of the primary spring;
- *k_S_*—stiffness of the secondary spring;
- *b_PV_*—damping coefficient of the primary vertical damper;
- *b_PY_*—damping coefficient of the primary yaw damper;
- *b_PL_*—damping coefficient of the primary lateral damper;
- *b_SV_*—damping coefficient of the secondary vertical damper.

### 4.2. A Description of a Track Model

The multibody model also contains a track model, on which the wagon runs during simulations. The track model includes the definition of a track geometry, rail profiles input files, track irregularities and other important parameters.

**Figure 8:**
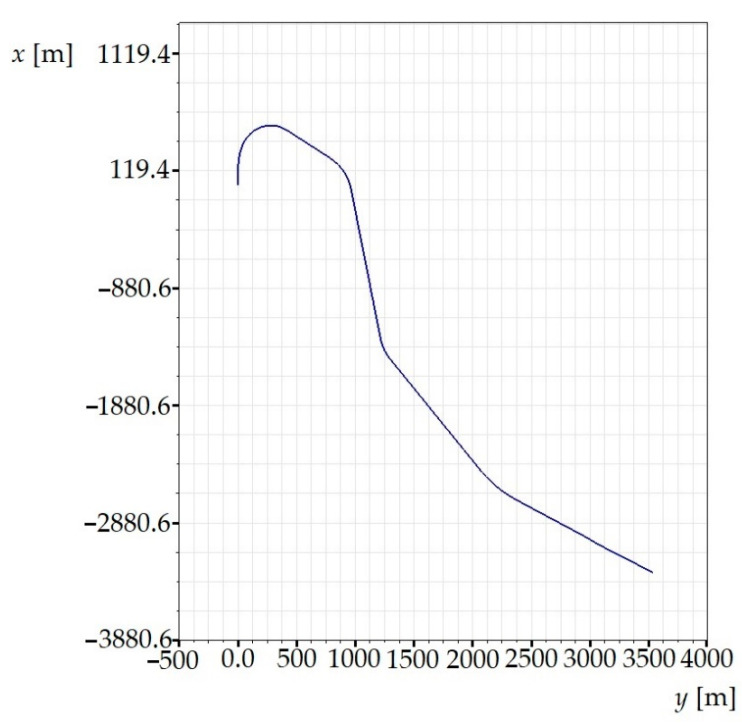
An illustration of the created railway track.

In our research, we have used a model of a real track section. The chosen track model is suitable for performing simulation computations and subsequent evaluations of ride comfort because it includes straight sections as well as sections with curves of various radii, i.e., sections with superelevation ramps and transient sections. The track gauge was 1435 mm, the rail cant 1:40 and the rail head profile UIC60. There parameters are prescribed in the source input file. An illustration of the track model is shown in Figure 8.

The track model also includes track irregularities. The definition of irregularities in the track model is important in terms of the excitation of the railway passenger car in order to simulate as real conditions as possible [64,65,66]. The track irregularities can be defined in the Simpack software as predefined stochastic irregularities according to the standard [67], either by means of the input file containing real measured irregularities or by means of a harmonic function, which is the simplest way. Our model includes track irregularities measured on the track. Thus, such a track model generates excitation of the vehicle and acceleration signals can be detected [68,69,70].

### 4.3. Simulation Analysis and Results

As described above, the main objective of this research is to investigate how the change of suspension parameters in the railway passenger car effects the ride comfort for passengers. Stiffness–damping parameters of springs and dampers, namely values of the parameters *k_P_*, *k_S_*, *b_PV_* and *b_SV_* (Figure 7), in the primary and secondary suspension systems were changed in three levels, at which both the ratio of stiffness of the secondary and primary springs as well as the ratio of damping of the vertical secondary and the vertical primary dampers were preserved. The damping coefficients of the rest of the dampers were unchanged. The characteristics of the springs were linear, while the characteristics of the dampers were non-linear.

The definition of the spring-damping parameters was based on the original parameters named as “Original”. The ratio of the stiffness *k_S_*/*k_P_* is 0.587. In the case of the dampers, an inclination of the tangent was changed, at which the ratio of the extreme values was preserved. Then, numerous combinations of *k_P_* and *k_S_*, as well as the characteristics of *b_PV_* and *b_VS_*, were defined and tested by means of simulations. This work presents only the finally chosen combinations of *k_P_* and *k_S_* and *b_PV_* and *b_SV_* in order to illustrate their effects on the ride comfort index *N_MV_*. These parameters are summarized in Table 3. The percentage of *k_P_*, *k_S_*, *b_PV_* and *b_SV_* expresses percentage by which the original value was changed. It was changed both to lower (sign “−”) and higher (sign “+”) values.

Simulations of running the railway passenger car were performed for various speeds. This section includes the resulting values of the ride comfort *N_MV_* distribution on the floor only for two selected speeds, namely for the speed of 60 km∙h^−1^ and 110 km∙h^−1^. These speeds were chosen as representative samples for evaluation of the acceleration signals, because a speed of 60 km∙h^−1^ is the average speed of trains on the track and the speed 110 km∙h^−1^ is the maximum speed at which trains can safely run through curves on a track.

**Figure 9:**
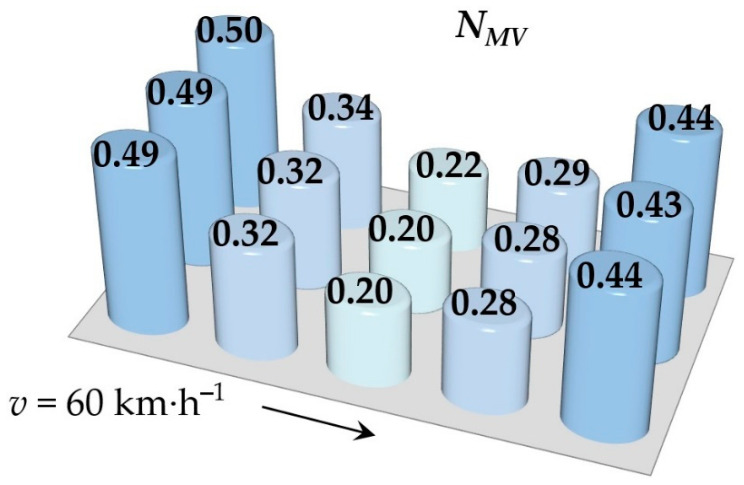
The distribution of the index *N_MV_* for a speed of 60 km∙h^−1^: the “Original” parameters.

**Figure 10:**
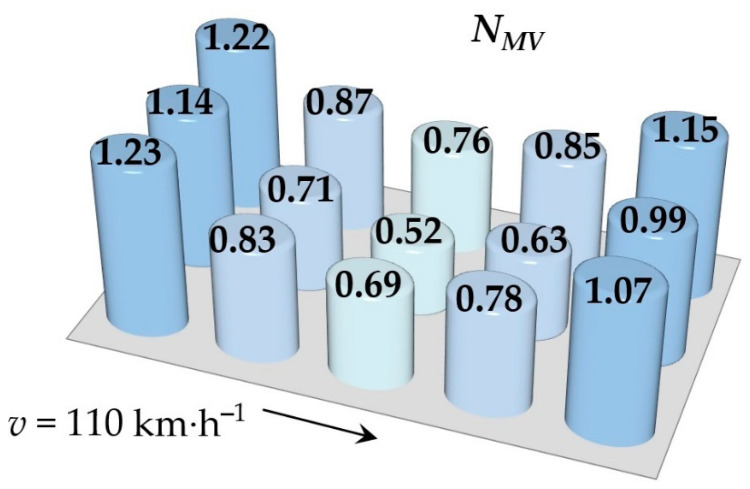
The distribution of the index *N_MV_* for a speed of 110 km∙h^−1^: the “Original” parameters.

**Figure 11:**
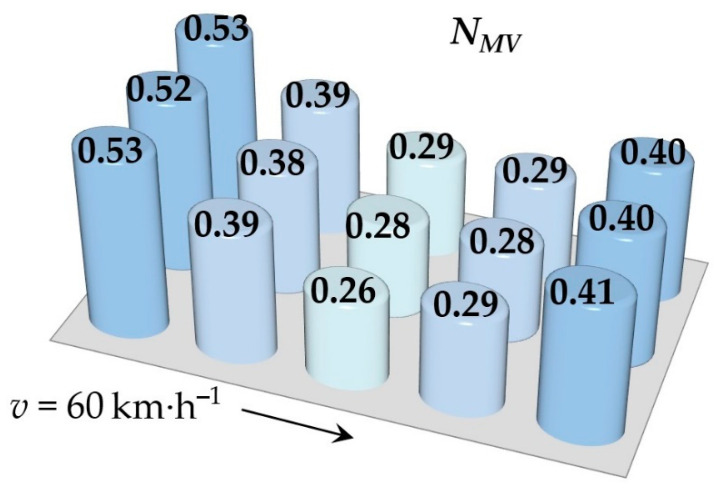
The distribution of the index *N_MV_* for a speed of 60 km∙h^−1^: the “Modification I” parameters.

**Figure 12:**
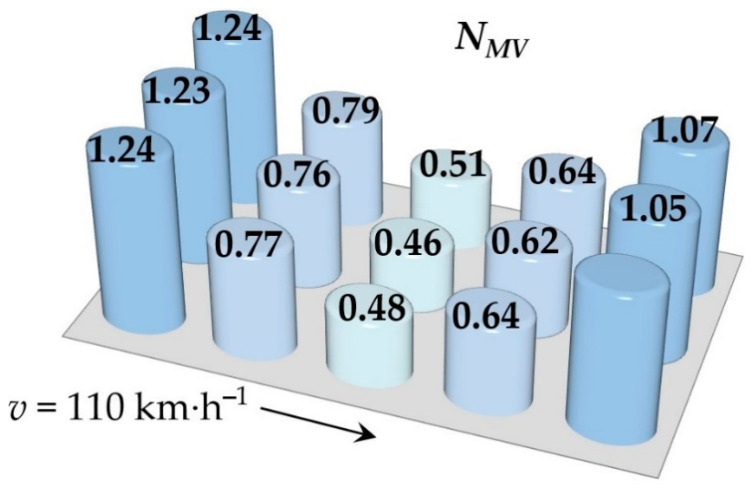
The distribution of the index *N_MV_* for a speed of 110 km∙h^−1^: the “Modification I” parameters.

**Figure 13:**
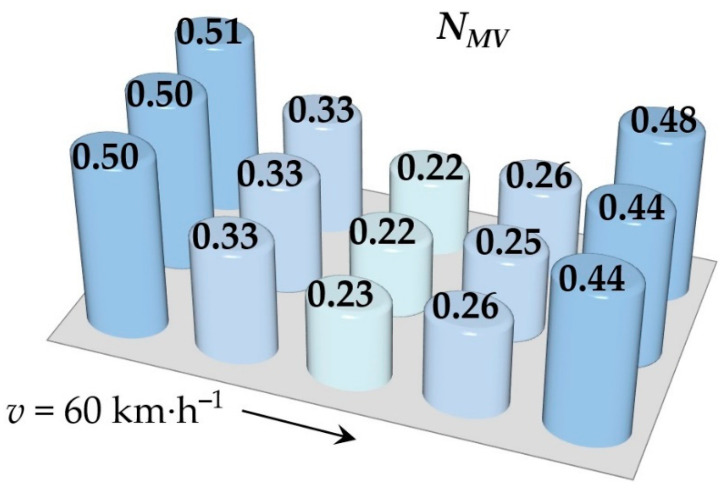
The distribution of the index *N_MV_* for a speed of 60 km∙h^−1^: the “Modification II” parameters.

**Figure 14:**
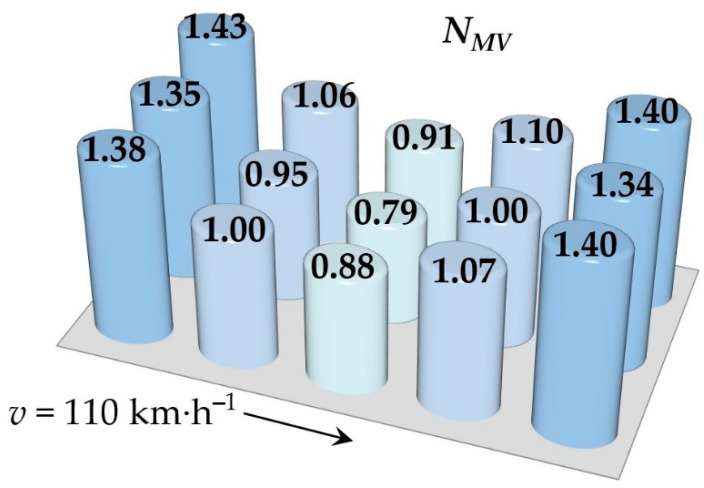
The distribution of the index *N_MV_* for a speed of 110 km∙h^−1^: the “Modification II” parameters.

Figure 9, Figure 10, Figure 11, Figure 12, Figure 13 and Figure 14 show the distribution of the ride comfort index *N_MV_* on the wagon body floor for various running conditions. These histograms are arranged according to the scheme shown in Figure 3.

The first two figures (Figure 9 and Figure 10) show the distribution of the ride comfort index *N_MV_* for the “Original” parameters of the suspension system (Table 3). Another two figures (Figure 11 and Figure 12) include the calculated results for the “Modification I” parameters (Table 3) when both the stiffness–damping coefficients of the primary suspension systems are softer and the stiffness–damping parameters of the secondary suspension system are stiffer. Finally, last two figures (Figure 13 and Figure 14) provide an overview of the distribution of the ride comfort index *N_MV_* for “Modification II” parameters, i.e., when the stiffness–damping parameters of the primary suspension are stiffer and the stiffness–damping parameters of the secondary suspension are softer. A description of the findings of the presented research are described in detail in the following section.

## 5. Discussion

The evaluation of the achieved results show that in the case of the railway passenger car running on the modelled real track, the ride comfort depends on the suspension parameters together with the running speed. A higher running speed leads to lower ride comfort. This is obvious for all three versions of the suspension parameters, i.e., for the “Original” (Figure 9 and Figure 10), for the “Modification I” (Figure 11 and Figure 12) as well as for the “Modification II” (Figure 13 and Figure 14) versions. It can be understood that higher running speeds causes greater excitations of the wagon body. Acceleration sensors detect greater values of acceleration, which are entered into the calculation of the ride comfort indices.

Our other findings include the values of the ride comfort indices. When we look at the results for the “Original” parameters, we can see that for both running speeds the values are under 1.5. This means that the ride is evaluated as very comfortable (Table 3). However, comparing these results for the chosen speed we can recognize that a higher running speed means a lower ride comfort.

The configuration “Modification I”, i.e., the softer suspension elements of the primary suspension system (both springs and dampers) and the stiffer suspension elements of the secondary suspension system (Figure 11 and Figure 12), lead to slightly higher values in the ride comfort indices *N_MV_* on the body floor at certain points and also indices of slightly lower values. This is recognized at both a speed of 60 km∙h^−1^ and a speed of 110 km∙h^−1^. These differences are not significant, and the ride can still be evaluated as very comfortable (Table 3).

The differences are more pronounced for the suspension parameters configuration “Modification II”, i.e., stiffer springs and dampers in the primary suspension and softer springs and dampers in the secondary suspension (Figure 13 and Figure 14). In case of the railway passenger car running at a speed of 60 km∙h^−1^, the resulting ride comfort indices are similar to the “Original” and “Modification I” configurations. However, the values of the ride comfort indices for the running speed of 110 km∙h^−1^ are higher and close to the limit of 1.5, which is a boundary between very comfortable and comfortable. It can be seen that higher values of the index *N_MV_* are calculated for all measured points on the body floor. This is interesting because the secondary suspension is intended mainly to ensure the comfort of passengers. This is because the softer springs and dampers in the secondary suspension leads to a greater tilt of the wagon body and thus accelerations in the *y* direction influence the resulting value of the *N_MV_* index. Despite the fact that this phenomenon could also be expected for the configuration “Modification I”, the achieved results do not confirm this. Another fact should be noted. The calculated ride comfort index *N_MV_* is primarily determined for evaluating the ride comfort on straight tracks. Also, our results suggest that the application of the ride comfort index *N_MV_* for evaluating ride comfort in curves has to be justified.

The presented results are so far only a part of more extensive and complex research focused on investigating the influence of the parameters of vehicles on ride comfort and running safety. It is important to assess the influence of the weight of the wagon body on the ride comfort. If the weight of the vehicle is higher, it can be expected that the values of the ride comfort index would be even higher due to greater centrifugal acceleration (*y* direction) as well as vertical (*z* direction) and longitudinal (*x* direction) acceleration.

Furthermore, springs with the suitable non-linear characteristics can be mounted. Such a suspension system is able to preserve similar (or even the same) waveforms of responses regardless of the weight of the vehicle.

Future research will be focused on evaluating the influence of changes in the parameters described above on ride comfort and safety. The principle applied in this research, which was used for a particular rail vehicle, can be also used in compliance with certain specifications in order to examine the characteristics of road vehicles, work machines, rescue vehicles and other similar vehicles.

## 6. Conclusions

Computer simulations of vehicle movement on a track represent a state of art way to investigate and evaluate their behavior in term of their dynamics.

The analysis of ride comfort for passengers remains a problem. The subjective experience of the comfort level which a passenger experiences in a vehicle can significantly affects their preference for a given means of a transport. In order to evaluate of ride comfort by means of simulation computations, the indirect method was used. This method is based on identifying the acceleration of the required points in different directions, their statistical processing and the calculation of a specific number (the comfort index).

In the presented research:

- The commercial simulation software Simpack was used;
- A virtual model of a railway passenger car was created;
- Passenger ride comfort was evaluated for the chosen parameters of the vehicle;
- The ride comfort index *N_MV_* on the body floor was calculated and graphs illustrating the *N_MV_* indices distributed on the body floor were depicted;
- It was concluded that the stiffness of the springs and damping coefficients of dampers of the secondary suspension system affected ride comfort more significantly than the stiffness–damping parameters of the primary suspension system.

## Figures and Tables

**Table 1:** Measured quantities and locations for evaluating the ride comfort of passengers by means of the index *N_MV_* [42].

Average Comfort, the Standard Method		
Ride Comfort Index	Measured Quantity	Measured Location
*N_MV_*	Accelerations in three directions (*x*, *y*, *z*)	Floor

**Table 2:** A scale for evaluating the ride comfort index *N_MV_* [42].

Value of the *N_MV_* Index	Level of the Ride Comfort
*N_MV_* < 1.5	Very comfortable
1.5 ≤ *N_MV_* < 2.5	Comfortable
2.5 ≤ *N_MV_* < 3.5	Medium comfortable
3.5 ≤ *N_MV_* < 4.5	Uncomfortable
*N_MV_* ≥ 4.5	Very uncomfortable

**Table 3:** A list of the parameters of the spring-damper elements.

		Stiffness		Damping	
Designation	Name	*k_P_*	*k_S_*	*b_PV_*	*b_SV_*
O	Original	0%	0%	0%	0%
I	Modification I	−45%	+45%	−45%	+45%
II	Modification II	+45%	−45%	+45%	−45%

## Data Availability

Not applicable.
